# A Comparative Study of Radiomics and Deep-Learning Based Methods for Pulmonary Nodule Malignancy Prediction in Low Dose CT Images

**DOI:** 10.3389/fonc.2021.737368

**Published:** 2021-12-17

**Authors:** Mehdi Astaraki, Guang Yang, Yousuf Zakko, Iuliana Toma-Dasu, Örjan Smedby, Chunliang Wang

**Affiliations:** ^1^ Department of Biomedical Engineering and Health Systems, KTH Royal Institute of Technology, Huddinge, Sweden; ^2^ Department of Oncology-Pathology, Karolinska Institutet, Stockholm, Sweden; ^3^ Cardiovascular Research Centre, Royal Brompton Hospital, London, United Kingdom; ^4^ National Heart and Lung Institute, Imperial College London, London, United Kingdom; ^5^ Imaging and Function, Radiology Department, Karolinska University Hospital, Solna, Stockholm, Sweden; ^6^ Department of Physics, Stockholm University, Stockholm, Sweden

**Keywords:** lung nodule, benign-malignant classification, lung cancer prediction, radiomics, deep classifier

## Abstract

**Objectives:**

Both radiomics and deep learning methods have shown great promise in predicting lesion malignancy in various image-based oncology studies. However, it is still unclear which method to choose for a specific clinical problem given the access to the same amount of training data. In this study, we try to compare the performance of a series of carefully selected conventional radiomics methods, end-to-end deep learning models, and deep-feature based radiomics pipelines for pulmonary nodule malignancy prediction on an open database that consists of 1297 manually delineated lung nodules.

**Methods:**

Conventional radiomics analysis was conducted by extracting standard handcrafted features from target nodule images. Several end-to-end deep classifier networks, including VGG, ResNet, DenseNet, and EfficientNet were employed to identify lung nodule malignancy as well. In addition to the baseline implementations, we also investigated the importance of feature selection and class balancing, as well as separating the features learned in the nodule target region and the background/context region. By pooling the radiomics and deep features together in a hybrid feature set, we investigated the compatibility of these two sets with respect to malignancy prediction.

**Results:**

The best baseline conventional radiomics model, deep learning model, and deep-feature based radiomics model achieved AUROC values (mean ± standard deviations) of 0.792 ± 0.025, 0.801 ± 0.018, and 0.817 ± 0.032, respectively through 5-fold cross-validation analyses. However, after trying out several optimization techniques, such as feature selection and data balancing, as well as adding context features, the corresponding best radiomics, end-to-end deep learning, and deep-feature based models achieved AUROC values of 0.921 ± 0.010, 0.824 ± 0.021, and 0.936 ± 0.011, respectively. We achieved the best prediction accuracy from the hybrid feature set (AUROC: 0.938 ± 0.010).

**Conclusion:**

The end-to-end deep-learning model outperforms conventional radiomics out of the box without much fine-tuning. On the other hand, fine-tuning the models lead to significant improvements in the prediction performance where the conventional and deep-feature based radiomics models achieved comparable results. The hybrid radiomics method seems to be the most promising model for lung nodule malignancy prediction in this comparative study.

## Introduction

**Figure 1:**
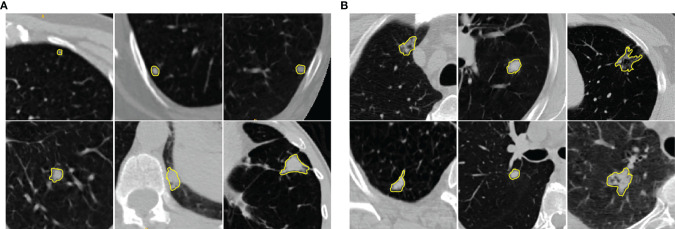
Illustration of **(A)** benign and **(B)** malignant pulmonary nodules in chest LDCT scans. The manually identified nodules are highlighted with yellow contours. The examples show that benign and malignant pulmonary nodules present similar visual characteristics. The cropped patches around the nodules (context images) provide the relative location of the nodules with respect to nearby structures.

Lung cancer is notoriously aggressive and accounts for the leading cause of cancer-related death worldwide (1). Early diagnosis of asymptomatic lung cancer plays a vital role in treatment planning that can significantly improve the survival rate of lung cancer patients (2). The National Lung Screening Trial (NLST), a large-scale trial involving more than 50000 individuals, has reported that screening with Low Dose Computed Tomography (LDCT) scans will result in a 20% of reduction in lung cancer mortalities (3). Most lung cancers emerge from small malignant pulmonary nodules that refer to moderately well-marginated round opacities with the largest diameter less than 3cm (4). Although most solitary pulmonary nodules have benign causes, 30%–40% of such nodules are malignant (5). In clinical practice, expert radiologists visually examine the CT volumes on a slice-by-slice basis and subjectively determine the likelihood of nodule malignancy that often yields to relatively high inter/intra-observer variability of the interpretations. Moreover, highly similar visual characteristics shared among benign and malignant pulmonary nodules make this manual assessment task even more challenging (see **Figure 1**). Therefore, it would be beneficial to develop Computer-Aided Diagnosis (CAD) tools to capture latent characteristics of the pulmonary nodules in order to assist the radiologist with the task of benign-malignant lung nodule classification.

In the context of CAD tools, many semi/fully automatic pulmonary nodule classification methods have been proposed in the literature. Among all the recent proposed solutions, radiomics analysis (6–10), and deep learning-based methods (11–15) render the most promising results. Strictly speaking, radiomics analysis aims at building predictive models based on extracting handcrafted features from lung nodules. These radiological image-based features are designed to quantify the latent characteristics of the medical images that are imperceptible to human eyes. On the other hand, deep learning approaches such as Convolutional Neural Networks (CNNs) are trained with an end-to-end scheme to automatically translate the input images into corresponding class labels by adaptively learning deep abstract features in the consecutive convolutional layers.

It has been shown that factors such as nodules’ size and heterogeneities in the intensity and textures of nodules are strongly associated with nodule malignancy (16). Radiomic features are often designed to capture such critical features from the nodule structures. The extracted radiomic features from lung nodules were employed to train learning algorithms such as logistic regression (17), linear discriminant analysis (18), random forests (19), and support vector machines (20) for malignancy identification. It has been shown that radiomic textural features are able to quantify the intra-tumor heterogeneities that appeared in CT volumes (21). In this context, several studies have investigated the ability of the textural features such as Gray Level Co-occurrence Matrix (GLCM), Gray Level Run Length Matrix (GLRLM), and Gray Level Zone Length Matrix (GLZLM) to distinguish malignant lung nodules from benign ones (17, 18, 20, 22). In addition, shape-based features were employed to quantify the morphological characteristics of lung nodules with irregular appearance (23, 24). Although each of the radiomics family can capture specific characteristics, their combination could cover different nodule attributes, and several promising results for lung cancer prediction have been reported (25–29).

Gaining from large-scale training image data, CNN models provide a uniform framework for jointly learning the hierarchical representative features extracted directly from the images and classification weights (30). Numerous 2D and 3D CNN networks have been developed for lung nodule classification tasks which were trained with either cropped volumetric patches or 2D slices extracted from multiple views (15, 31, 32). To conquer the challenges of small-scale CT images and the small size of the lung nodules, an Agile-CNN model was proposed based on a hybrid setting of conventional AlexNet and LeNet networks and achieved competitive classification performance (33). To further improve the classification power, recent methods rely on ensemble learning in which multiple different deep learning models are developed, and their outcomes are integrated into a single classification model (34). In this context, Xu et al. (14) employed three shallow 3D networks trained with multi-scale cropped CT volumes to preserve contextual information. By further modifying the training procedure and objective function, they achieved a malignancy prediction score of 0.94 in terms of Area Under the Receiver Operating Characteristic Curve (AUROC) in an unbalanced dataset. To make the learned deep features interpretable, Lei et al. (15) developed a Soft Activation Mapping (SAM) to enable the analysis of fine-grained lung nodule features with a CNN model and then combined the high-level deep features with SAM to improve the classification accuracy to 0.99. Xie et al. (35) developed a semi-supervised adversarial classification model that consists of an unsupervised adversarial autoencoder network, a supervised classification network, and learnable transition layers to integrate both labeled and unlabeled CT volumes and achieved a classification accuracy of 0.92.

Both deep learning and radiomics have shown great potential to identify lung nodule malignancy in CT volumes and resulted in comparable performance in different datasets. However, there is a lack of conclusive evidence to support one type of method being better than the other. Most of the previous studies were done on relatively small datasets, which may not be considered a fair comparison as it is known that deep-learning based methods require a greater number of training images to achieve optimal performance. Also, many of those studies were performed on private datasets, which does not allow validation by other groups. This study aims to present an objective comparison among a series of carefully selected conventional radiomics methods, end-to-end deep learning models, and deep-feature based radiomics pipelines for pulmonary nodule malignancy prediction on an open database that consists of 1297 manually delineated lung nodules (both source code and annotation labels will be made publicly available upon acceptation). In addition to the prediction models, we also investigated the complementary role of the context region of lung nodules. Finally, a hybrid model was developed by pooling the extracted radiomic features and learned deep features to assess their compatibility with respect to nodule malignancy prediction. Finally, optimization steps such as feature selection methods and balancing the class labels were applied on radiomics, deep features, and their combination to improve the discrimination power.

## Materials and Methods

The investigated models consist of three major components: a radiomics module, a deep learning module, and a hybrid module. The *radiomics module* incorporates the radiomics analysis starting with feature extraction, followed by feature engineering steps, and finally, building predictive models. The *deep learning module* consists of the development of a dual-pathway CNN model to predict nodule malignancy by simultaneously training nodule target and nodule context images. The deep learning module was investigated thoroughly in our previous research (36) on the same dataset; therefore, in the current study, we adopt the results of already examined models. Finally, the *hybrid module* represents the approach of pooling the radiomic features and deep features together.

### Experimental Data

The Kaggle Data Science Bowl 2017 (37) contains a total number of 2101 clinical chest LDCT scans from which 1397, 198, and 506 subjects belong to the training, validation, and test sets, respectively. The objective of this challenge was to automatically predict lung cancer status; for that, each image was labeled as “1” if the patient was diagnosed with lung cancer within one year from the scan and “0” otherwise. The challenge organizer provided only the target labels of the training set at the patient level, and the validation/test labels are not accessible anymore as the challenge platform is closed. It should be noted that additional information, such as nodule segmentation masks, associated clinical data, and laboratory examinations, was not supplied.

In this study, out of 1397 training scans, 968 LDCT volumes were manually inspected by an expert radiologist, which led to the delineation of 1297 pulmonary nodules for further analyses. In addition to the segmentation masks, image patches that best cover each nodule context were cropped and extracted. For each nodule, visual radiological features including “cavitation with thick/thin wall”, “attached to the artery/fissure/pleura”, “calcification or fat content”, “dragging the pleura”, “spiculation” as well as “size >3cm” were extracted. **Figure 1** in **Supplementary Material** indicates the distribution of the studied nodules.

### Image Pre-Processing

Prior to radiomic feature extraction, all the cropped patches were preprocessed in three steps. First, original patches were resampled isotropically to a unified inner plane spacing as 0.2*mm* ^3^ using a bicubic interpolation function. Then the intensity ranges were clamped to [-1000,500] in terms of Hounsfield Units. A further step was applied only for deep learning module analyses in which the image patch was rescaled by zero-padding the original sizes into 128×128×128 voxels followed by intensity normalization in the range of [0,1].

### Radiomics Analysis

**Figure 2:**
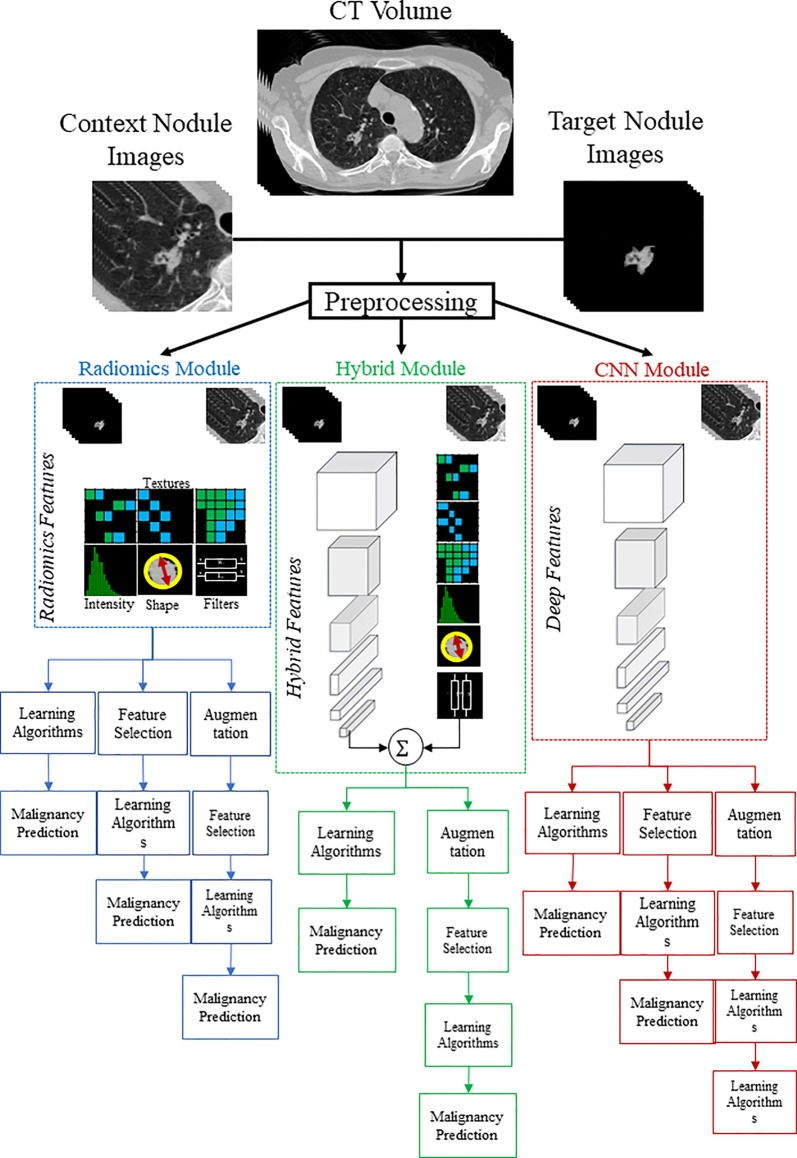
Graphical demonstration of the study pipeline. To predict lung nodule malignancy, three modules were studied. In the CNN module (red color), deep networks were trained with context and target nodule images separately and simultaneously. In the Radiomics module (blue color), handcrafted features were extracted from both target and context nodule images to train the learning algorithms. In the hybrid module (green color), extracted radiomic features were combined with learned deep features to form the hybrid sets.

We adopted the radiomics descriptors to quantify the geometric, intensity, and textural characteristics of the nodules by extracting a total number of 1334 3D descriptors using the standard PyRadiomics package (38). Specifically, 14 geometric, 18 First-Order Statistics (FOS), and 70 Second-Order Statistics (SOS) features were extracted from the target nodules. In addition, FOS and SOS features were also extracted from multi-scale transformed nodules filtered with Wavelet and Laplacian of Gaussians (38) (**Table 1** in **Supplementary Material**). The mentioned features were extracted from both target nodules and context images to capture intra-nodule characteristics as well as nodule context attributes (**Figure 2**- Radiomics Module).

As irrelevant or partially relevant features can adversely impact the classification performance, and in order to minimize the risk of overfitting, filter-based Feature Selection (FS) methods, as well as a wrapper FS method, were employed. In particular, filter-based FS algorithms include (1) Constant (CST): removing constant features; (2) Correlation (Corr): removing linearly related features; (3) Mutual Information (MI): removing nonlinearly related features; (4) RELevance in Estimating Features (RELIEF): estimating the quality of the features based on how well the features can distinguish the subjects that are close to each other; and (5) Least Absolute Shrinkage and Selection Operator (LASSO): applying coefficients to the features and shrink to zero those features which are less predictive (39). On the other hand, Forward Feature Selection (FFS) as a wrapper FS method was adopted to evaluate the performance of the learning algorithms in different combinations of feature subsets. In addition, Principal Component Analysis (PCA) was employed as well to transform the high-dimensional feature set into a lower dimension (26). The prediction power of the selected features was then evaluated with 8 different learning algorithms: Adaptive Boosting (Adab), Decision Tree (DT), Random Forest (RF), K-Nearest Neighbor (KNN), Support Vector Machines (SVM), Linear/Quadratic Discriminant Analysis (LDA/QDA), as well as Naïve Bayesian (**Table 1**).

**Table 1:** The prediction power of the radiomic features extracted from target nodule images with different learning algorithms and feature selection methods over the balanced dataset.

Learning Algorithm	Target Radiomic Prediction Performance (AUROC)							
	*Feature Selection*							
	*None*	*CST*	*Corr*	*LASSO*	*RELIEF*	*MI*	*PCA*	*FFS*
Adab	**0.889 ± 0.016**	**0.863** ± **0.021**	**0.881** ± **0.031**	**0.864** ± **0.026**	0.671 ± 0.019	0.531 ± 0.037	**0.868** ± **0.015**	**0.911** ± **0.016**
DT	0.723 ± 0.011	0.733 ± 0.027	0.703 ± 0.019	0.711 ± 0.028	0.642 ± 0.013	0.523 ± 0.024	0.712 ± 0.026	0.730 ± 0.032
RF	0.871 ± 0.008	0.849 ± 0.025	0.846 ± 0.028	0.856 ± 0.023	**0.765** ± **0.018**	0.517 ± 0.031	0.862 ± 0.026	0.891 ± 0.011
KNN	0.850 ± 0.016	0.846 ± 0.016	0.807 ± 0.036	0.833 ± 0.017	0.735 ± 0.021	0.671 ± 0.089	0.846 ± 0.017	0.870 ± 0.023
SVM	0.777 ± 0.011	0.774 ± 0.029	0.752 ± 0.025	0.775 ± 0.027	0.751 ± 0.020	0.522 ± 0.040	0.775 ± 0.028	0.802 ± 0.008
LDA	0.655 ± 0.045	0.680 ± 0.032	0.785 ± 0.017	0.75 ± 0.027	0.741 ± 0.031	0.735 ± 0.018	0.771 ± 0.028	0.796 ± 0.011
QDA	0.778 ± 0.172	0.696 ± 0.181	0.747 ± 0.016	0.738 ± 0.024	0.753 ± 0.031	**0.840** ± **0.020**	0.752 ± 0.026	0.865 ± 0.006
Naive	0.763 ± 0.006	0.759 ± 0.023	0.742 ± 0.030	0.731 ± 0.022	0.756 ± 0.034	0.583 ± 0.046	0.739 ± 0.024	0.808 ± 0.010

For each feature selection algorithm, the highest value is marked in bold.

### End-to-End Deep Learning Model

In our previous study on the same dataset (36), we proposed a dual pathway network architecture to train both the nodule target images and nodule context images simultaneously in a unified network. This unified network consists of two convolutional pathways for representation learning, each followed by a few dense layers and a shared final dense layer. In other words, while the nodule target pathway is assumed to mainly learn the association between the intra-nodule representations and class labels, the role of the nodule context pathway is primarily to learn the correlations between the context information and the class labels. Therefore, having concatenated the learned features from each of the pathways in a last shared dense layer, the model is enforced to predict the class labels by adaptively learning the intra- and context-nodules attributes simultaneously. Different supervised models, including VGG (40), ResNet (41), DenseNet (42), EfficientNet (43), and a variational autoencoder (44) as an unsupervised model, were employed for the convolutional backbones. Moreover, the conventional single pathway models were trained with either nodule target or nodule context images separately to extract features from the corresponding regions. More details of the studied method can be found in (36).

### Deep Feature Extraction

In addition to end-to-end training of the deep networks, the learned deep features from each network were extracted to train a separate learning algorithm. In specific, for the dual pathway models, 2048 deep features were extracted from the last but one dense layer representing the target and context nodule attributes. Moreover, with the same approach,1024 deep features were extracted from the single pathway networks for each of the nodule target and nodule context images. The extracted deep features were then analyzed by different learning algorithms. Experimentally, random forest was selected as the learning algorithm to train the extracted deep features after feature augmentation, as it consistently led to more stable results than the other classifiers (36).

### Nodule Classification

Radiomic features and deep features were used separately to distinguish the benign nodules from malignant ones. In addition, the same task was done by combining the extracted deep features with radiomic features in a hybrid model. In particular, the extracted radiomic features from target nodule images were analyzed both independently and in combination with deep features learned from the target nodule images. Similar analyses were conducted by combining the features extracted and learned from the context nodule images. Finally, to examine how context and target nodule images would complement each other, the prediction power of a combination of target nodule features and context nodule features was investigated as well.

From 1297 studied lung nodules, 876 cases belong to the benign group, and 421 nodules are labeled as malignant. Such an unequal distribution of the class labels leads to a high bias toward the majority class and, in turn, degrades the prediction power of the learning algorithms and will result in poor prediction of the minority class. To tackle this issue, we employed Synthetic Minority Oversampling Technique (SMOTE) (45) to synthesize new samples from the minority class. In specific, SMOTE fits a hypercube among some instances in the feature space of malignant nodules to interpolate new samples. Balancing the dataset with SMOTE yields the generation of 455 new instances belong to the malignant nodule class, which increased the total number of studied data to 1752. All the analyses were performed with a 5-fold cross-validation fashion, and the performance of the model was assessed by using the Area Under the Receiver Operating Characteristic Curve (AUROC) metric calculated as averages of corresponding cross-validation folds. Finally, to test statistical significances between different experiments, a pairwise AUROC comparison method proposed by Delong et al. (46) was employed. **Figure 2** shows a graphical illustration to visualize the feature analysis workflow for all the examined modules.

## Results

In this section, the performance of the radiomic features for lung nodule malignancy prediction is presented and compared against the prediction power of deep learning models. In addition, the classification power of the hybrid feature pools is quantified as well.

### Handcrafted Radiomics

Radiomics analysis was performed by using 1334 3D quantitative descriptors with and without feature selection methods over 8 distinct learning algorithms. The analyses were conducted over the original imbalanced dataset and augmented, balanced set as well. Comparing the results between the balanced (**Tables 1**–**3**) and imbalanced datasets (**Tables 2–4** in **Supplementary Material**), one can observe that synthesizing new samples in the feature space from the minority class resulted in a remarkable improvement in classification performance. For instance, the prediction power of the Adab learning algorithm without any FS method before and after augmenting the data are AUROC_unbalanced_ = 0.779 and AUROC_balanced_ = 0.889, respectively.

**Table 2:** The prediction power of the radiomic features extracted from context nodule images with different learning algorithms and feature selection methods over the balanced dataset.

Learning Algorithm	Context Radiomic Prediction Performance (AUROC)							
	*Feature Selection*							
	*None*	*CST*	*Corr*	*LASSO*	*RELIEF*	*MI*	*PCA*	*FFS*
Adab	**0.895** ± **0.007**	**0.867** ± **0.022**	**0.866** ± **0.020**	**0.871** ± **0.009**	0.580 ± 0.017	0.643 ± 0.078	**0.852** ± **0.009**	**0.916** ± **0.011**
DT	0.718 ± 0.011	0.697 ± 0.025	0.695 ± 0.015	0.702 ± 0.032	0.571 ± 0.021	0.550 ± 0.039	0.697 ± 0.031	0.744 ± 0.027
RF	0.881 ± 0.008	0.843 ± 0.024	0.855 ± 0.009	0.864 ± 0.011	0.645 ± 0.025	0.613 ± 0.044	0.845 ± 0.007	0.901 ± 0.014
KNN	0.852 ± 0.007	0.824 ± 0.019	0.811 ± 0.010	0.843 ± 0.021	0.625 ± 0.015	0.590 ± 0.023	0.827 ± 0.019	0.779 ± 0.029
SVM	0.777 ± 0.012	0.757 ± 0.010	0.689 ± 0.010	0.716 ± 0.008	0.685 ± 0.012	0.571 ± 0.020	0.715 ± 0.009	0.817 ± 0.023
LDA	0.682 ± 0.040	0.727 ± 0.018	0.774 ± 0.014	0.758 ± 0.017	0.743 ± 0.017	0.746 ± 0.022	0.751 ± 0.016	0.842 ± 0.027
QDA	0.841 ± 0.013	0.705 ± 0.033	0.777 ± 0.032	0.751 ± 0.025	**0.770** ± **0.012**	**0.863** ± **0.067**	0.739 ± 0.024	0.872 ± 0.010
Naive	0.767 ± 0.014	0.690 ± 0.006	0.757 ± 0.029	0.745 ± 0.009	0.682 ± 0.010	0.609 ± 0.038	0.728 ± 0.020	0.820 ± 0.013

For each feature selection algorithm, the highest value is marked in bold.

**Table 3:** The prediction power of the joint context and target radiomic with different learning algorithms and feature selection methods over the balanced dataset.

Learning Algorithm	Combined Radiomic Prediction Performance (AUROC)							
	*Feature Selection*							
	*None*	*CST*	*Corr*	*LASSO*	*RELIEF*	*MI*	*PCA*	*FFS*
Adab	**0.908** ± **0.014**	**0.883** ± **0.021**	**0.883** ± **0.016**	**0.888** ± **0.014**	0.570 ± 0.043	0.676 ± 0.005	0.876 ± 0.014	**0.921** ± **0.010**
DT	0.739 ± 0.032	0.699 ± 0.018	0.728 ± 0.011	0.720 ± 0.014	0.568 ± 0.030	0.594 ± 0.020	0.702 ± 0.016	0.772 ± 0.013
RF	0.897 ± 0.016	0.858 ± 0.032	0.877 ± 0.019	0.865 ± 0.028	0.620 ± 0.045	0.621 ± 0.021	**0.880** ± **0.011**	0.910 ± 0.008
KNN	0.872 ± 0.014	0.844 ± 0.014	0.804 ± 0.013	0.860 ± 0.007	0.650 ± 0.018	0.604 ± 0.029	0.848 ± 0.012	0.816 ± 0.028
SVM	0.756 ± 0.023	0.711 ± 0.019	0.709 ± 0.035	0.722 ± 0.022	0.625 ± 0.038	0.574 ± 0.574	0.724 ± 0.022	0.818 ± 0.022
LDA	0.711 ± 0.027	0.726 ± 0.012	0.802 ± 0.023	0.759 ± 0.011	0.730 ± 0.019	0.647 ± 0.032	0.768 ± 0.012	0.827 ± 0.020
QDA	0.862 ± 0.014	0.711 ± 0.015	0.827 ± 0.028	0.766 ± 0.020	**0.736** ± **0.017**	**0.903** ± **0.007**	0.744 ± 0.010	0.887 ± 0.015
Naive	0.783 ± 0.023	0.702 ± 0.020	0.741 ± 0.019	0.731 ± 0.024	0.628 ± 0.020	0.544 ± 0.013	0.736 ± 0.021	0.825 ± 0.021

For each feature selection algorithm, the highest value is marked in bold.

In addition, comparing **Tables 1**, **2** reveals the fact that target nodule and context nodule images represent different nodule characteristics that carry distinct prediction powers. However, as can be seen in **Table 3**, combining the target nodule features with context features improved the accuracy and yielded the highest classification power in radiomics analyses. Among the employed learning algorithms, Adab method embedded on decision trees yielded the highest prediction power when feature selection methods were not applied. Comparing the performance of the models after applying different feature selection methods, one can infer that FFS method consistently improved the prediction power and outperformed other feature selections except the two cases of KNN and QDA. Accordingly, the highest predictive value of the target radiomics set was achieved from a subset of features selected with FFS and trained with Adab classifier (AUROC = 0.911), which accounts for an improvement of 2.2 percent from the same classifier but without feature selection. Similar behavior was observed in context radiomics where integrating FFS into the Adab classifier improved the discrimination power from 0.895 to 0.916. The classification accuracy was even more improved from 0.908 to 0.921 when the combination of context and target radiomics were analyzed with the same method.

### End-to-End Deep Learning Models

**Table 4** shows a summary of the best results achieved by the deep learning-based analyses, which were published in our previous study (36). The first column represents the prediction power achieved by end-to-end training of the networks on the imbalanced datasets. In other words, for each of the target nodule, context nodule, and their combined (dual-pathway) images, 5 different networks were trained, and the best performance for each nodule image type is reported in **Table 4**. Comparing the performance of the end-to-end deep networks trained with the target and context nodule images, one can infer that context features were more informative than target nodules. Furthermore, similar to radiomics, integrating the target and context nodule images into a unified network resulted in a slightly higher prediction power. In fact, the AUROC values of dual-pathway models outperformed each of the single pathway models trained with context and target images separately. These enhancements imply that the two distinct image types can become complementary. In other words, the combined features achieved the highest AUROC value by virtue of the joint use of context and target deep features that can adequately complement the intricate characterizations of shape, intensity, and textural heterogeneity of the nodules.

**Table 4:** The prediction power of the deep learning-based analyses.

Feature Type	Deep Features Prediction Performance (AUROC)	
	*End-to-end training*	*Feature augmentation*
Target nodule	0.801 [0.777,0.824]	0.906 [0.890,0.921]
Context nodule	0.806 [0.788,0.827]	0.927 [0.912,0.940]
Combined	0.824 [0.798,0.837]	0.936 [0.921,0.950]

The combined model refers to a dual-pathway network that was fed by context and target nodule images simultaneously. Lower and upper limits of confidence interval at 95% level are indicated in square brackets.

### Deep Features

The last column of **Table 4** indicates the results of the deep feature-based radiomics pipeline. Strictly speaking, after training the networks, the imbalanced learned deep features were extracted from one to last dense layer of each model and augmented after applying the SMOTE algorithm. The balanced deep features were then used to train an RF model. More details over the deep feature analyses and the effects of employed different optimization techniques on the prediction powers of deep features were examined and reported in our previous study (36).

Comparing the first column of **Table 4** with **Tables 2**–**4** in **Supplementary Material** indicates that end-to-end training the deep classifiers with the unbalanced dataset could predict the class labels of the lung nodules more accurately than radiomics analyses over the imbalanced feature set without applying the FS methods. For instance, while integrating the imbalanced target and context radiomic features led to achieving an AUROC of 0.773 with the RF learning algorithm, end-to-end training a dual-pathway model resulted in an AUROC value of 0.824 that accounts for 5.1% of improvement. Besides, significant improvements were achieved when the extracted deep features were employed in a radiomics-based pipeline. Specifically, applying the SMOTE algorithm to the unbalanced extracted deep features could successfully boost the discrimination power of the RF model by up to 12.1% (*AUROC_context-imbalanced_* = 0.806 vs. *AUROC_context-balanced_* = 0.927). In addition, balancing the deep features seems to be more constructive than balancing the radiomic features in terms of discrimination power. In particular, AUROC values achieved by the RF model on balanced deep features outperformed the performance of the RF model trained with balanced radiomic features with rather significant margins. (*AUROC_target_*: 0.906 *vs.* 0.891, *AUROC_context_*: 0.927 *vs.* 0.901; and *AUROC_combined_*: 0.936 *vs.* 0.910).

### Hybrid Feature Analysis

**Figure 3:**
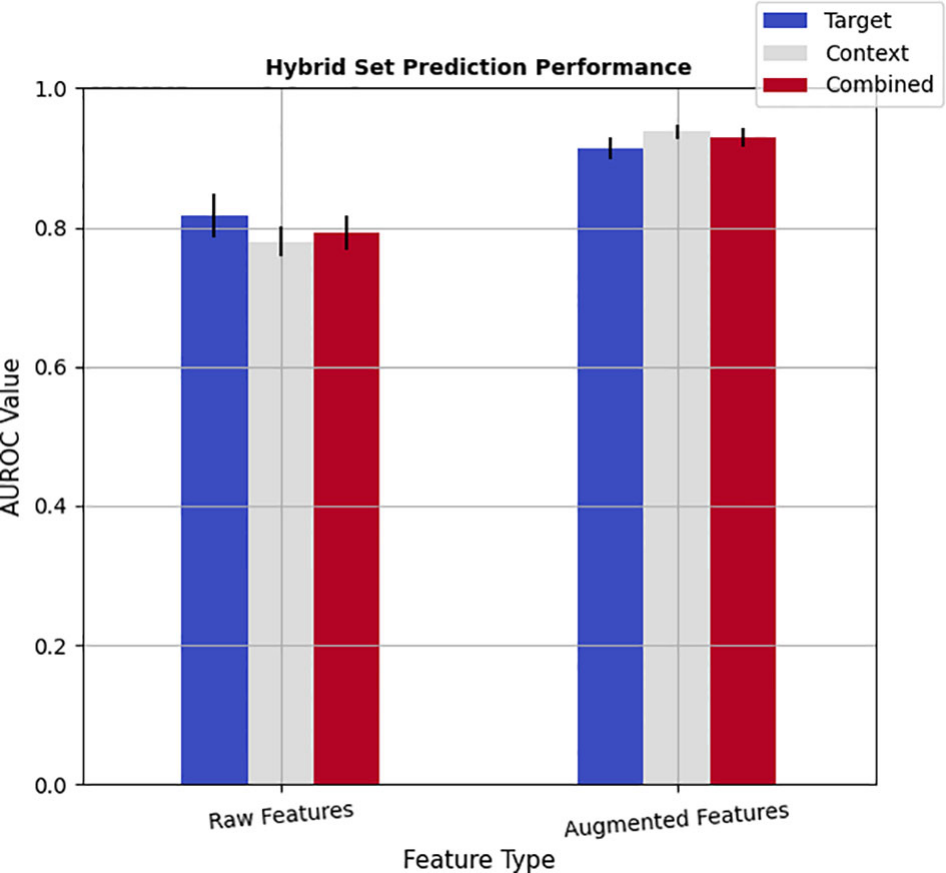
The prediction power of the hybrid model: combinations of deep and radiomic features.

To investigate whether the learned deep features would complement radiomics descriptors, a hybrid model was developed by pooling the radiomics and deep features into a mixture set. Accordingly, for each of the target, context, and combined radiomics pools, corresponding learned features from single-pathways and dual pathway deep networks were merged for both the raw features and augmented features (see **Figure 3**). It should be noted that assessing the capability of hybrid feature pools was performed by employing the Adab learning algorithm integrated with the FFS method because the highest prediction performance of the radiomics was achieved by this method. The results show that incorporating the imbalanced deep features into imbalanced radiomics could slightly improve the performance of imbalanced radiomics alone by up to 1.9% (*AUROC_radiomics_combined_*: 0.774 *vs. AUROC_hybrid_combined_*: 0.793 *vs.* 0.891). However, this combination was not led to enriching the performance of the end-to-end deep learning methods. On the other hand, noticeable improvements were observed in the performance of the hybrid models after augmenting both the radiomics and deep features. In particular, merging the balanced deep features with balanced radiomics not only successfully improved the prediction power of radiomics alone but also slightly enhanced the performance of deep features as well. These improvements were observed for both target and context nodule images. In addition, the balanced hybrid feature pools of combined target and context features resulted in an AUROC value of 0.929, which is superior to the combined radiomic features alone with an AUROC value of 0.921. Besides, even with the hybrid model, context pools were more representative than the target pools, which is in line with both the radiomics and deep feature pools individually. Last but not least, the best prediction accuracy (AUROC=0.938) among all the analyses was achieved by a hybrid model in which the balanced context radiomics merged with the balanced deep features learned from context images which point to the complementary role of context radiomics and context deep features. **Table 8** in **Supplementary Material** indicates the pairwise statistical comparison between radiomic features and deep features. Finally, **Table 9** in **Supplementary Material** shows the same statistical assessment between target, context, and combined feature sets in the hybrid module.

### Feature Importance and Class Separability

**Figure 2** in **Supplementary Material** demonstrates the top 20 important features that contributed to explaining the class labels when training the RF classifier for different sets of radiomics, and hybrid feature sets. As can be seen, textural features cast the majority of the important features in radiomics analysis in both the target and context nodules. However, the contribution of shape-based features in context radiomics indicates the fact that shape descriptors could be involved in quantifying the context information of the regions covered the nodules. The same figure for the hybrid sets illustrates that most of the important attributes in the hybrid target set derived from radiomics features, while deep features contributed more in the hybrid context set. Moreover, the most informative radiomic features identified from FS methods are reported in **Table 5** in the **Supplementary Material**.

**Figure 3** in **Supplementary Material** shows the scatter plots of the class separability of different feature pools. In practice, T-distributed Stochastic Neighbor Embedding (TSNE) (47) statistical method was employed to nonlinearly reduce the dimensionality of the feature space and visualize the distribution of the data points based on their similarity in a 2D space. Because the dimension of the feature sets was larger than 1000, we first applied the PCA to project the feature sets into a 70D space and then apply the TSNE method. Interestingly, the class separability of deep features is more obvious than radiomics. These scatter plots are consistent with the quantitative results reported in **Tables 1**, **2**, **4**, and **Figure 3**.

**Figure 4:**
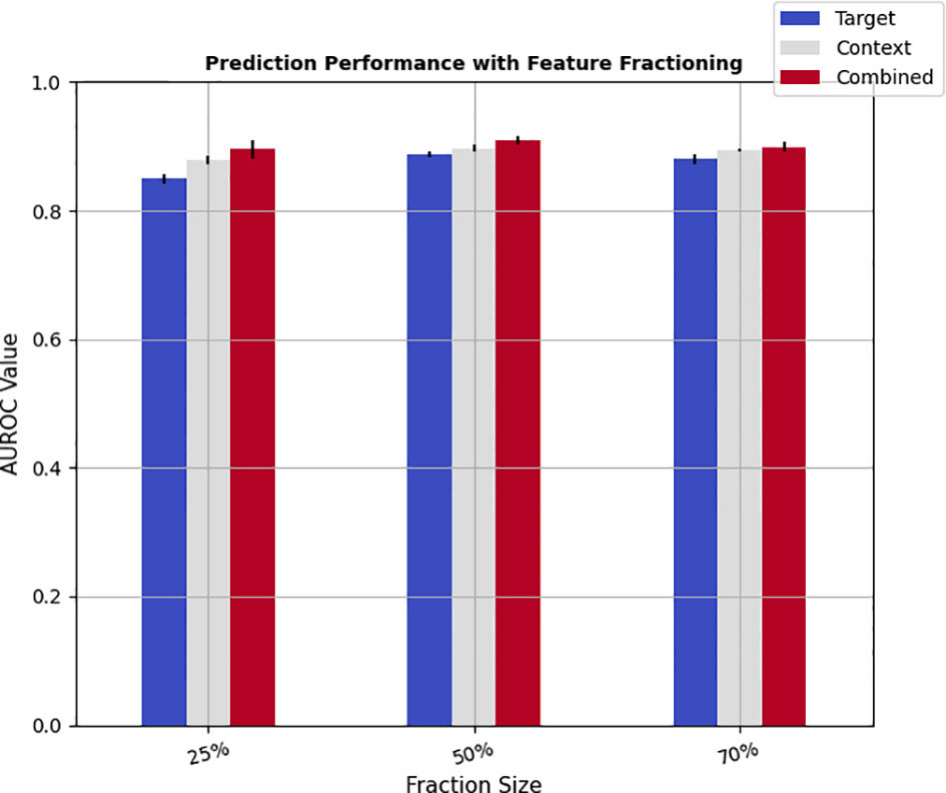
Effect of training size on the prediction power of the hybrid feature sets.

Finally, to study the impact of the size of training samples on the prediction performance, the feature pools were split randomly into training and test subsets with varying proportions. In specific, the learning algorithm was trained with 25%, 50%, and 70% of the feature pools as training samples, while the rest of 75%, 50%, and 30% were dedicated to test sets. The discrimination scores were then calculated on the test set by applying a 5-fold cross-validation scheme. **Figure 4** shows the results of feature fractioning achieved from the hybrid feature pools, and **Tables 6, 7** in **Supplementary Materials** demonstrate the same evaluations calculated from radiomics and deep features separately. As was expected, increasing the size of the training samples leads to higher prediction powers. Besides, the discrimination powers of different fractions of deep features were higher than those of the radiomic features, while the performance on fractions of hybrid sets and deep features were relatively close. Such results are in agreement with the prediction scores reported in **Figure 3**.

### Conventional Radiomics vs. End-to-End Deep Learning Model vs. Deep-Feature-Based Radiomics

In this study, four different strategies, including conventional radiomics analysis, end-to-end deep learning classifiers, deep feature based radiomics as well as the combination of radiomics and deep features, were investigated to predict lung nodule malignancy. Moreover, the effect of fine-tuning steps such as feature augmentation and feature selection on the model performance were examined as well. To better compare the performance of each method, **Table 5** shows a summary of the discrimination powers. In this table, ‘fine-tuning’ refers to the feature augmentation step, and ‘context-info’ indicates whether the context nodule images were included or not. Accordingly, the best results achieved by analyzing the radiomic features are presented in ‘best conventional radiomics’ column, and the best performance achieved by end-to-end training of deep learning models are showed in ‘best end-to-end deep learning model’ column. In addition, the extracted deep features from the learned networks were analyzed with the radiomics pipeline, i.e., feature selection and feature augmentation steps were applied and followed by training the learning algorithms. The best performance of such analyses is summarized in ‘best deep-feature-based radiomics’ column. Finally, an overview of the prediction powers achieved by concatenating the radiomic features with the extracted deep features is shown in the last column of the table ‘best hybrid model’.

**Table 5:** Comparing the prediction power of the employed methods.

Prediction Performance Comparison (AUROC)					
*Fine-tuning*	*Context-info*	*Best conventional radiomics*	*Best end-to-end deep learning model*	*Best deep-feature-based radiomics*	*Best hybrid model*
Before	No	0.792 ± 0.025	0.801 [0.777,0.824]	0.753 [0.743,0.775]	0.817 ± 0.032
After	No	0.911 ± 0.016	–	0.906 [0.890,0.921]	0.914 ± 0.015
Before	Yes	0.777 ± 0.017	0.806 [0.788,0.827]	0.761 [0.736,0.779]	0.780 ± 0.022
After	Yes	0.916 ± 0.011	0.824 [0.798,0.837]	0.927 [0.912,0.940]	0.929 ± 0.013

Note that the “best end-to-end deep learning model” column presents the performance of two single pathway models trained with target and context nodule images separately and one dual pathway model trained with both target and context images simultaneously. Lower and upper limits of confidence interval at 95% level are indicated in square brackets.

## Discussion

The separation of benign from malignant pulmonary nodules on chest LDCT scans is an important step toward the early detection of lung cancers which in return offer the best chance for cure. In clinical practice, this vital step is done manually by expert radiologists on a slice by slice basis. However, the possibility of operator bias on one side, and the presence of highly similar visual characteristics shared between the benign and malignant nodules on the other side, can potentially lessen the accuracy of manual nodule classification. Therefore, many computer-aided models for automatic/semi-automatic classification of pulmonary nodules have been developed as assistant tools to facilitate such a demanding task. In a general view, these models can be categorized into two groups: handcrafted radiomics and end-to-end deep learning models. In this study, we conducted a comprehensive comparison of the performance of radiomics and deep learning models for lung nodule malignancy prediction on a relatively large-scale dataset consists of 1297 manually delineated lung nodules. In addition, we applied several optimization steps on both extracted radiomic features and learned deep features to improve the prediction performance. In this context, to reliably distinguish intra-nodule characteristics from nodule contextual attributes, both radiomics and deep features were extracted from target nodule images and context nodule images. Moreover, in order to efficiently capture the critical nodule characteristics such as shape, intensity, and textural heterogeneities, a hybrid feature set was constructed by pooling deep and radiomic features together.

The high correspondence between intra-nodule heterogeneity and malignancy alludes to the privilege of textural radiomic features. From 1334 radiomic descriptors, almost 73% of them represent textural features from the original and multi-scale filtered images. Interestingly, having performed the radiomics analysis with/without FS methods, textural features contributed significantly to classification results. In fact, a majority of the informative features without FS and a majority of the selected features with the FFS method come from textural families, which is in agreement with the reported results by other studies (30, 32, 48). Performing the filter-based FS methods on radiomics has not always yielded the improvement of the prediction power. In other words, statistically less correlated feature subset selected from Corr, LASSO, RELIEF, and MI methods were not necessarily informative with respect to the class labels and therefore lessened the prediction power in some cases. On the other hand, the greedy FFS method reduces the dimensionality of radiomic pools by selecting a combination of feature subsets with the highest prediction performance. In addition, as was expected, combining multiple weak classifiers into a single robust learning algorithm, the Adab method outperformed the rest of the classifiers in terms of prediction power (49). As the underlying training procedures of the RF classifier, to some extent, are close to that of Adab, it achieved the second strong prediction power. Moreover, 35% of the features selected from FFS are repeated between these two classifiers (**Table 5** in **Supplementary Material**). Furthermore, fractioning the radiomics training set to even 25% of all the features resulted in satisfactory prediction power (*AUC*_25%_= 0.827 vs. *AUC*_80%_= 0.889). While the target nodule images hold intra-nodule attributes such as intensity and textural heterogeneities, shape and morphological features, as well as size-based properties, the context nodule images include the characteristics of surrounding tissues as well. Such differences in the image contents led to slightly higher prediction performance for context images. These results are in agreement with clinical practices in which the expert radiologists not only focus on the visual attributes of the nodules but also closely examine the contextual information around the nodules by inspecting the same nodule in three different orthogonal views in several prior and succeeding slices. The important features which contributed to malignancy prediction in context nodule images consist of several shapes and FOS-based features instead of focusing only on textural radiomics, which implies the fact that the model could capture the changes in the geometry and intensity heterogeneities in the nodule images after including context information. In addition, the observed improvements in the prediction power of combined nodule features point to the complementary role of nodule target and nodule context images. Of course, it should be noticed that applying the FS methods could reduce the risk of overfitting that would occur after combining context and target nodule features. More importantly, employing the SMOTE as an augmentation technique could successfully hinder the classification bias toward the majority class by balancing the class labels, which in return improved the classification accuracy significantly in all the radiomics experiments. In general, our analyses show that radiomic features are capable of quantifying the differences between challenging benign and malignant pulmonary nodules and have a great potential to achieve promising results.

Similar to radiomics, augmented deep context features outperformed the augmented deep target features, and their combination improved the accuracy of malignancy prediction as well. Training end-to-end deep classifiers could perform slightly better than radiomics analyses for each of target nodule images, context nodule images, and their combination. This can be described by the fact that end-to-end training of the networks enforces the models to learn to extract the features with the highest correspondence with respect to the class labels. On the other hand, radiomic features are extracted regardless of the class labels so that they would need further carefully designed processing steps to maximize their discrimination power. Besides that, augmenting the deep features by synthesizing new data points in the feature space to balance the class labels effectuates a terrific improvement in prediction powers. It is noteworthy that employing the conventional strategies such as including an equal number of both class labels in each batch of images during the model training may be helpful to avoid biasing toward the majority class. However, they often would not be able to dramatically boost the prediction performance. In general, although the deep learning models outperformed the radiomics analyses when the mentioned feature selection and augmentation techniques were not applied, we can infer that exerting such fine-tuning steps results in comparable performance.

The classification improvement achieved from integrating deep features with radiomics points to the fact that the hybrid descriptors could successfully cover a wide range of nodule characteristics from quantitative textures to abstract shape features. Such observed improvements are in agreement with other studies performed on lung nodule classification tasks (30, 32). Although radiomic descriptors are designed inspired by radiological quantitative imaging features, CNN models are trained to capture abstract features with high relevancy with respect to the class labels. Nevertheless, fusing these two distinct feature set in order to enhance the prediction power should be conducted carefully. In other words, simply aggregating the two feature sets will dramatically increase the length of the features and potentially increase the risk of overfitting. Such larger feature sets can even exacerbate the problem of class imbalance. Additionally, the careless fusion of these feature sets would increase the number of correlated features, which in return would afflict the performance of the models. The inferior performance of the raw hybrid feature sets can be caused by the described reasons. However, fine-tuning the hybrid feature sets by balancing the classes and reduce the dimensionality of the features by employ the proper FS methods can be considered as beneficial strategies to efficiently gain from the hybrid feature pools that resulted in the highest prediction power.

In this comparative study, we examined the two most conventional approaches of radiomics and deep learning methods to predict pulmonary nodule malignancy in LDCT images. Despite the promising results achieved and compared in this paper, in our future studies, we aim to test the proposed approach on external datasets both as inference models and transfer learning strategies. In addition, we plan to investigate other fusion methods to integrate radiomic descriptors into end-to-end deep learning models efficiently.

## Conclusion

We conducted a comparative study to distinguish benign pulmonary nodules from malignant nodules in LDCT images. To do so, we performed a comprehensive radiomics analysis by investigating the prediction power of 1334 extracted radiomic features trained with 8 learning algorithms integrated with 7 feature selection methods. We compared the radiomics performance against several deep classifiers trained on the same datasets. We examined the effect of optimization strategies such as synthesizing the feature points to balance the class labels, extracting features from context images, and combine context features with features extracted from target nodule images. Our results suggest that effective integration of radiomics and deep features improves the performance of nodule classification and produces more accurate results.

## Data Availability Statement

The original contributions presented in the study are included in the article/**Supplementary Material**. Further inquiries can be directed to the corresponding author. The implementations can be found at https://github.com/Astarakee/Radiomics_pipeline.

## Author Contributions

MA: Conceptualization, Methodology, Formal analysis, Writing - original draft, Visualizations. CW: Conceptualization, Methodology, Writing - review & editing, Supervision, Project administration. YZ: data annotation, Writing - review & editing. IT-D: Writing - review & editing, Supervision. GY: Writing - review & editing. ÖS: Writing - review & editing, Supervision, Resources, Funding acquisition. All authors contributed to the article and approved the submitted version.

## Funding

This study was supported in part by the Swedish Childhood Cancer Foundation [grant no. MT2016-0016], in part by the Swedish innovation agency Vinnova [grant no. 2017-01247], and in part by the Swedish Research Council (VR) [grant no. 2018-04375]. Guang Yang was supported in part by the British Heart Foundation [Project Number: TG/18/5/34111, PG/16/78/32402], the European Research Council Innovative Medicines Initiative [DRAGON, H2020-JTI-IMI2 101005122], the AI for Health Imaging Award [CHAIMELEON, H2020-SC1-FA-DTS-2019-1 952172], and the UK Research and Innovation Future Leaders Fellowship [MR/V023799/1].

## Conflict of Interest

The authors declare that the research was conducted in the absence of any commercial or financial relationships that could be construed as a potential conflict of interest.

## Publisher’s Note

All claims expressed in this article are solely those of the authors and do not necessarily represent those of their affiliated organizations, or those of the publisher, the editors and the reviewers. Any product that may be evaluated in this article, or claim that may be made by its manufacturer, is not guaranteed or endorsed by the publisher.
