# Supplementary material for: A Comparative Study of Radiomics and Deep-Learning Based Methods for Pulmonary Nodule Malignancy Prediction in Low Dose CT Images
Source: Front Oncol. 2021 Dec 17;11:737368. doi: 10.3389/fonc.2021.737368 (PMC8718670; doi:10.3389/fonc.2021.737368)
Supplement: Supplementary file 1 [file DataSheet_1.pdf]

Supplementary Material

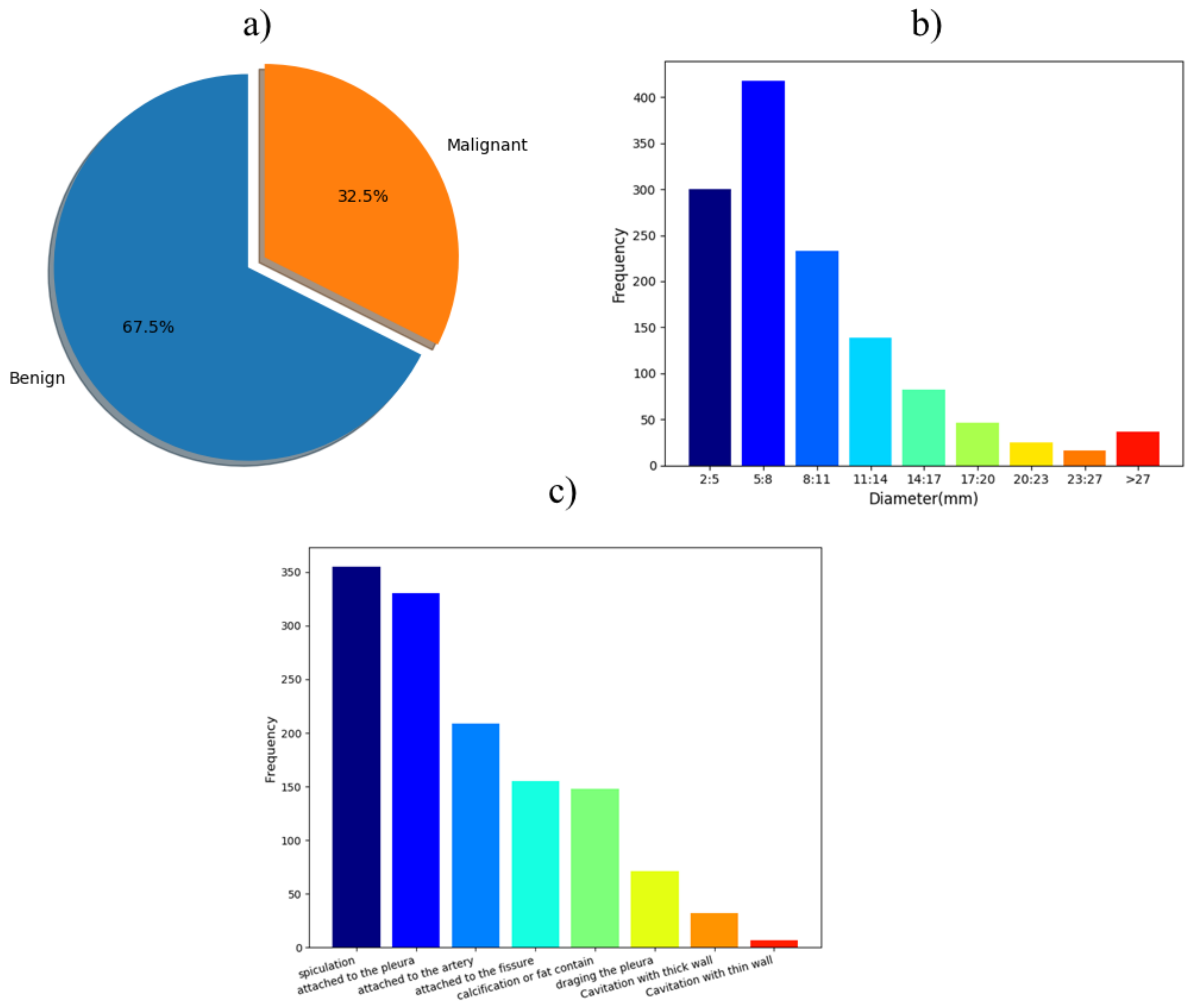

Figure 1. Distribution of the 1297 segmented lung nodules; a) proportion of the lung nodules with respect to class labels; b) frequency of the nodules in terms of nodules diameter; c) frequency of the nodules with respect to radiological attributes.

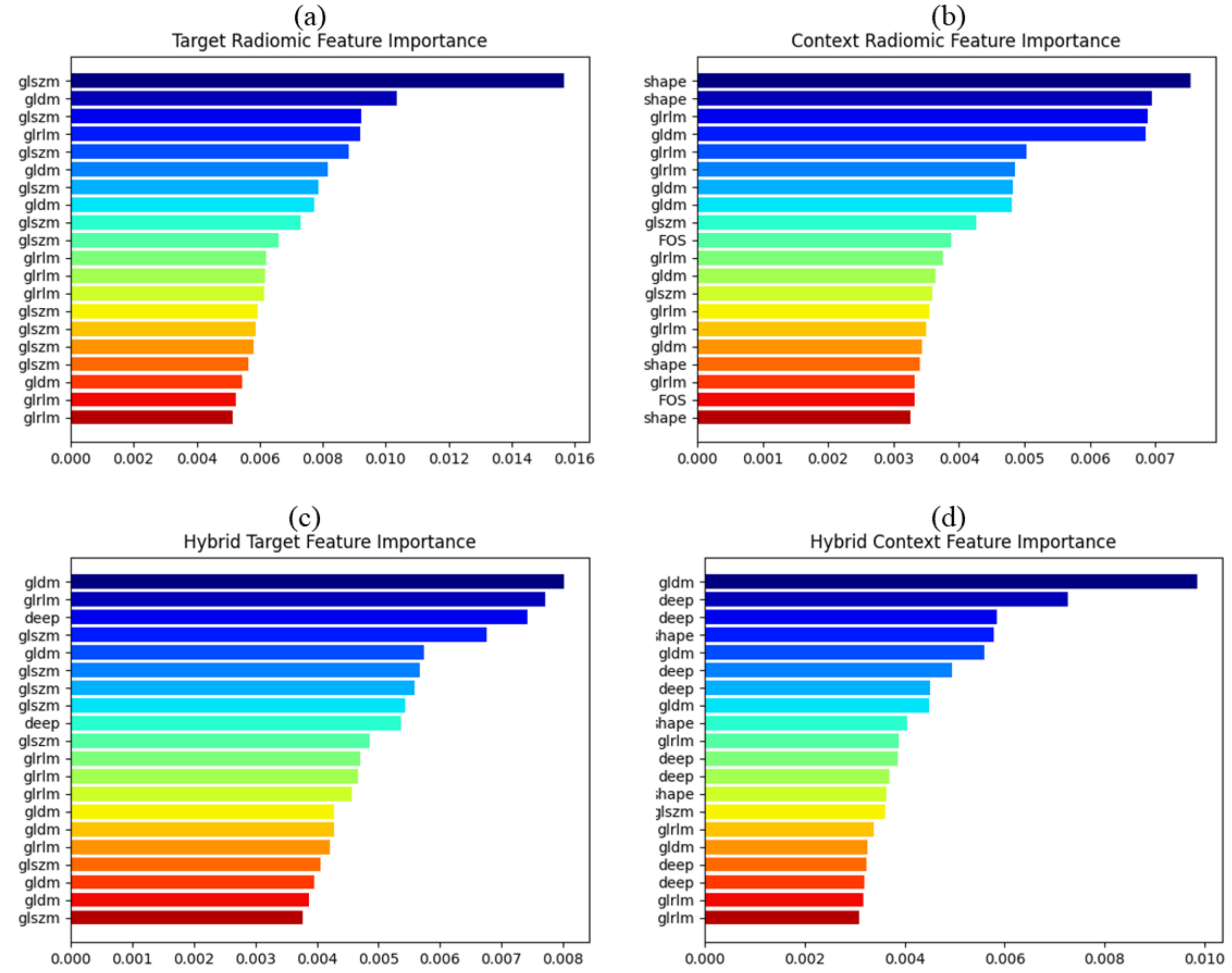

Figure 2. Illustration of the top 20 important features contributed to the prediction. Plots (a) and (b) show the features with significant contribution in the target radiomics and context radiomics, respectively. Plots (c) and (d) represent the important features of hybrid target and hybrid context feature sets, respectively.

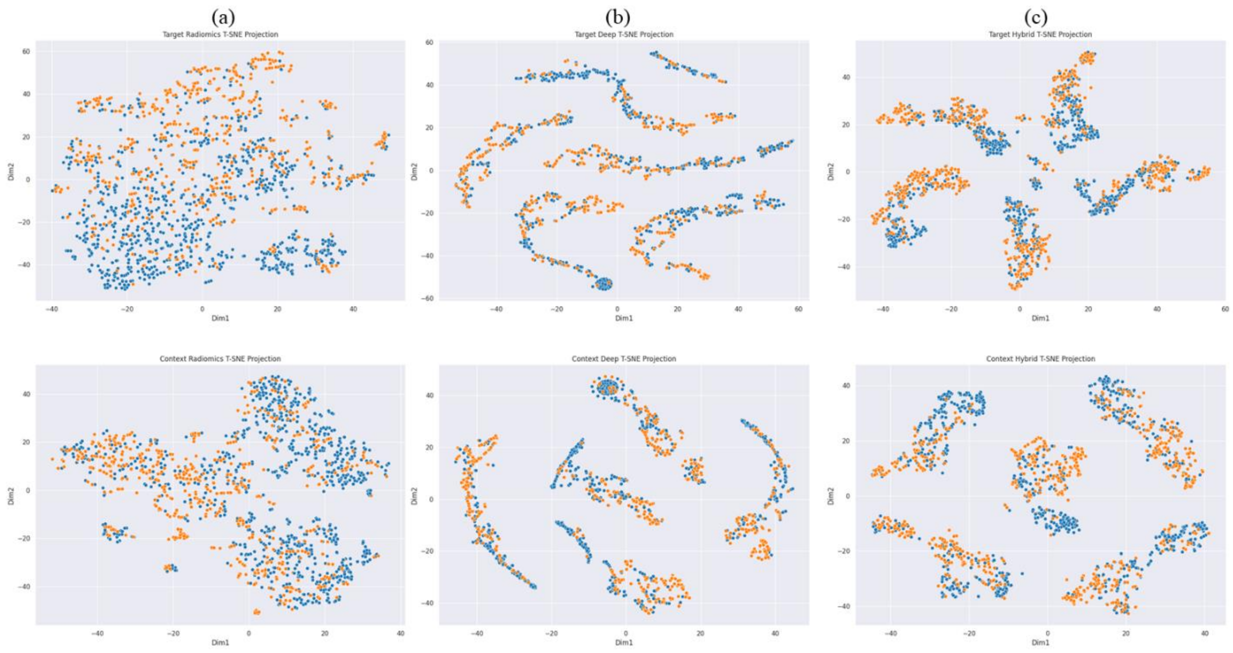

Figure 3. 2D visualization of feature space with respect to the class labels after nonlinearly reduced the dimensionalities by PCA and TSNE. Columns (a), (b), and (c) show the scatter plots corresponding to radiomics, deep, and hybrid feature pools. The first row shows the features extracted and learned from target nodule and the second row demonstrates the context nodule based features.

Table 1. Extracted radiomic features.

Radiomic Features		
Category	***# of features***	***Names***
First-order statistics	18	10Percentile, 90Percentile, Energy, Entropy, InterquartileRange, Kurtosis, Maximum, MeanAbsoluteDeviation, Mean,Median, Minimum, Range, RobustMeanAbsoluteDeviation, RootMeanSquared, Skewness, TotalEnergy, Uniformity, Variance
Geometric	14	Elongation, Flatness, LeastAxisLength, MajorAxisLength, Maximum2DDiameterColumn, Maximum2DDiameterRow, Maximum2DDiameterSlice, Maximum3DDiameter, MeshVolume, MinorAxisLength, Sphericity, SurfaceArea, SurfaceVolumeRatio, VoxelVolume
Gray level co-occurrence matrix	24	Autocorrelation, ClusterProminence, ClusterShade, ClusterTendency, Contrast, Correlation, DifferenceAverage, DifferenceEntropy, DifferenceVariance,Id, Idm, Idmn, Idn, Imc1, Imc2, InverseVariance, JointAverage, JointEnergy, JointEntropy,MCC, MaximumProbability, SumAverage, SumEntropy, SumSquares
Gray level run-length matrix	16	GrayLevelNonUniformity, GrayLevelNonUniformityNormalized, GrayLevelVariance, HighGrayLevelRunEmphasis, LongRunEmphasis, LongRunHighGrayLevelEmphasis, LongRunLowGrayLevelEmphasis, LowGrayLevelRunEmphasis, RunEntropy, RunLengthNonUniformity, RunLengthNonUniformityNormalized, RunPercentage, RunVariance, ShortRunEmphasis, ShortRunHighGrayLevelEmphasis, ShortRunLowGrayLevelEmphasis
Gray level size-zone matrix	16	GrayLevelNonUniformity, GrayLevelNonUniformityNormalized, GrayLevelVariance, HighGrayLevelZoneEmphasis, LargeAreaEmphasis, LargeAreaHighGrayLevelEmphasis, LargeAreaLowGrayLevelEmphasis, LowGrayLevelZoneEmphasis, SizeZoneNonUniformity, SizeZoneNonUniformityNormalized, SmallAreaEmphasis, SmallAreaHighGrayLevelEmphasis, SmallAreaLowGrayLevelEmphasis, ZoneEntropy, ZonePercentage, ZoneVariance
Gray level dependence matrix	14	DependenceEntropy, DependenceNonUniformity, DependenceNonUniformityNormalized, DependenceVariance, GrayLevelNonUniformity, GrayLevelVariance, HighGrayLevelEmphasis, LargeDependenceEmphasis, LargeDependenceHighGrayLevelEmphasis, LargeDependenceLowGrayLevelEmphasis, LowGrayLevelEmphasis, SmallDependenceEmphasis, SmallDependenceHighGrayLevelEmphasis, SmallDependenceLowGrayLevelEmphasis
Multi-scale Wavelet	704	First-order statistics and textural features were extracted from 8 levels of Wavelet decompositions
Multiscale Laplacian of Gaussian (LoG)	528	First-order statistics and textural features were extracted from LoG images with Gaussian radius varied from 0.5 to 3 (0.5 increments)

Table 2. The prediction power of the radiomic features extracted from target nodule images with different learning algorithms and feature selection methods over the unbalanced dataset. For each feature selection algorithm, the highest value is marked in bold.

**Learning Algorithm**	**Target Nodule Radiomic Prediction Performance (AUC)**							
	Feature Selection							
	None	*CST*	*Corr*	*LASSO*	*RELIEF*	*MI*	*PCA*	*FFS*
Adab	0.779±0.032	0.766±0.033	0.752±0.025	0.746±0.028	0.626±0.015	0.611±0.035	0.755±0.035	0.823±0.016
DT	0.631±0.027	0.620±0.032	0.594±0.032	0.613±0.026	0.604±0.029	0.568±0.015	0.619±0.033	0.677±0.046
RF	**0.792±0.025**	**0.788±0.031**	**0.770±0.025**	0.761±0.027	0.765±0.027	0.592±0.051	**0.772±0.032**	**0.825±0.025**
KNN	0.756±0.020	0.744±0.026	0.725±0.030	0.748±0.030	0.714±0.023	0.600±0.059	0.744±0.028	0.792±0.021
SVM	0.779±0.027	0.778±0.031	0.762±0.032	**0.779±0.032**	0.763±0.025	0.570±0.041	0.749±0.031	0.771±0.031
LDA	0.577±0.022	0.555±0.018	0.763±0.035	0.773±0.034	**0.770±0.038**	**0.720±0.036**	0.762±0.031	0.801±0.020
QDA	0.518±0.020	0.512±0.016	0.714±0.029	0.768±0.035	0.752±0.038	0.626±0.026	0.753±0.029	0.808±0.024
Naive	0.765±0.033	0.765±0.033	0.753±0.030	0.760±0.034	0.761±0.041	0.574±0.032	0.749±0.038	0.795±0.018

Table 3. The prediction power of the radiomic features extracted from context nodule images with different learning algorithms and feature selection methods over the unbalanced dataset. For each feature selection algorithm, the highest value is marked in bold.

**Learning Algorithm**	**Context Nodule Radiomic Prediction Performance (AUC)**							
	Feature Selection							
	None	*CST*	*Corr*	*LASSO*	*RELIEF*	*MI*	*PCA*	*FFS*
Adab	0.761±0.023	0.657±0.034	0.685±0.026	0.690±0.038	0.574±0.045	0.590±0.055	0.672±0.030	0.772±0.025
DT	0.609±0.039	0.565±0.035	0.555±0.022	0.583±0.039	0.547±0.026	0.558±0.031	0.564±0.020	0.666±0.020
RF	**0.777±0.017**	**0.680±0.035**	**0.713±0.053**	**0.713±0.057**	0.586±0.043	0.595±0.059	**0.711±0.037**	0.794±0.044
KNN	0.746±0.022	0.651±0.020	0.672±0.043	0.672±0.024	0.592±0.022	0.548±0.019	0.661±0.025	0.700±0.027
SVM	0.776±0.016	0.670±0.049	0.683±0.033	0.707±0.050	0.677±0.052	0.549±0.030	0.694±0.049	0.799±0.029
LDA	0.542±0.056	0.657±0.035	0.682±0.052	0.701±0.041	**0.722±0.067**	**0.644±0.033**	0.725±0.043	**0.818±0.031**
QDA	0.523±0.027	0.677±0.037	0.674±0.032	0.706±0.039	0.718±0.053	0.499±0.001	0.701±0.035	0.788±0.024
Naive	0.762±0.013	0.673±0.036	0.681±0.021	0.702±0.042	0.670±0.056	0.561±0.057	0.686±0.044	0.798±0.031

Table 4. The prediction power of the joint target and context radiomic features with different learning algorithms and feature selection methods over the unbalanced dataset. For each feature selection algorithm, the highest value is marked in bold.

**Learning Algorithm**	**Combined Nodule Radiomic Prediction Performance (AUC)**							
	Feature Selection							
	None	*CST*	*Corr*	*LASSO*	*RELIEF*	*MI*	*PCA*	*FFS*
Adab	0.762±0.010	0.748±0.012	0.752±0.024	0.759±0.030	0.482±0.029	0.620±0.034	0.718±0.033	**0.845±0.011**
DT	0.652±0.012	0.616±0.020	0.585±0.012	0.628±0.017	0.480±0.032	0.552±0.025	0.603±0.037	0.687±0.047
RF	0.773±0.022	**0.750±0.019**	**0.761±0.022**	**0.776±0.020**	0.568±0.022	**0.662±0.059**	**0.741±0.038**	0.826±0.012
KNN	0.691±0.026	0.680±0.027	0.665±0.028	0.693±0.052	0.561±0.026	0.615±0.058	0.690±0.035	0.770±0.022
SVM	**0.774±0.015**	0.706±0.033	0.672±0.041	0.707±0.035	0.600±0.029	0.618±0.078	0.714±0.035	0.795±0.016
LDA	0.555±0.021	0.720±0.037	0.738±0.013	0.741±0.037	**0.694±0.026**	0.567±0.045	0.734±0.041	0.821±0.016
QDA	0.541±0.027	0.696±0.051	0.693±0.037	0.724±0.040	0.689±0.026	0.507±0.007	0.712±0.044	0.828±0.024
Naive	0.734±0.020	0.692±0.034	0.689±0.012	0.718±0.035	0.639±0.018	0.594±0.042	0.692±0.035	0.814±0.014

Table 5. The 20 most informative radiomic features identified with forward feature selection method integrated within different learning algorithms.

**Learning Algorithms**	**Feature subset**
Adab	original_shape_Flatness, original_firstorder_Energy,original_glcm_ClusterProminence<br>log-sigma-0-5-mm-3D_firstorder_RootMeanSquared, log-sigma-0-5-mm-3D_gldm_HighGrayLevelEmphasis<br>log-sigma-1-mm-3D_firstorder_Range, log-sigma-1-mm-3D_glszm_LargeAreaEmphasis<br>log-sigma-1-5-mm-3D_firstorder_Mean, log-sigma-2-mm-3D_glszm_LargeAreaEmphasis<br>log-sigma-2-5-mm-3D_firstorder_Energy, log-sigma-2-5-mm-3D_glszm_LargeAreaHighGrayLevelEmphasis<br>log-sigma-3-mm-3D_firstorder_TotalEnergy, wavelet-LLH_glrlm_RunLengthNonUniformity<br>wavelet-LHL_firstorder_Energy, wavelet-HHL_glszm_LargeAreaHighGrayLevelEmphasis<br>wavelet-HHH_glszm_LargeAreaHighGrayLevelEmphasis, wavelet-LLL_firstorder_RootMeanSquared<br>wavelet-LLL_firstorder_Variance, wavelet-LLL_gldm_HighGrayLevelEmphasis<br>wavelet-LLL_gldm_SmallDependenceHighGrayLevelEmphasis
DT	original_glcm_ClusterTendency, original_glrlm_GrayLevelNonUniformity<br>original_gldm_GrayLevelNonUniformity, log-sigma-0-5-mm-3D_glcm_Autocorrelation<br>log-sigma-0-5-mm-3D_glrlm_GrayLevelNonUniformity, log-sigma-1-5-mm-3D_firstorder_Minimum<br>log-sigma-1-5-mm-3D_gldm_DependenceNonUniformity, log-sigma-1-5-mm-3D_gldm_GrayLevelNonUniformity<br>log-sigma-2-mm-3D_glrlm_GrayLevelNonUniformity, log-sigma-2-mm-3D_glszm_LargeAreaEmphasis<br>log-sigma-2-5-mm-3D_firstorder_10Percentile, log-sigma-2-5-mm-3D_glcm_ClusterShade<br>wavelet-LHL_glrlm_LongRunHighGrayLevelEmphasis, wavelet-HLL_firstorder_Variance<br>wavelet-HLL_glcm_ClusterProminence, wavelet-HLL_gldm_LargeDependenceEmphasis<br>wavelet-LLL_firstorder_10Percentile, wavelet-LLL_firstorder_MeanAbsoluteDeviation<br>wavelet-LLL_glrlm_GrayLevelNonUniformity
RF	original_firstorder_Energy, log-sigma-1-mm-3D_firstorder_Variance, log-sigma-1-mm-3D_glszm_ZoneVariance<br>log-sigma-2-mm-3D_firstorder_Range, log-sigma-2-mm-3D_firstorder_TotalEnergy<br>log-sigma-2-mm-3D_firstorder_Variance, log-sigma-2-mm-3D_glszm_LargeAreaEmphasis<br>log-sigma-2-5-mm-3D_firstorder_Energy, log-sigma-2-5-mm-3D_firstorder_Range<br>log-sigma-2-5-mm-3D_glrlm_GrayLevelNonUniformity<br>log-sigma-2-5-mm-3D_glszm_LargeAreaHighGrayLevelEmphasis<br>log-sigma-2-5-mm-3D_gldm_LargeDependenceHighGrayLevelEmphasis<br>log-sigma-3-mm-3D_firstorder_TotalEnergy, log-sigma-3-mm-3D_glcm_ClusterProminence<br>wavelet-LLH_firstorder_TotalEnergy, wavelet-LHL_firstorder_Energy,wavelet-HLL_firstorder_Energy

	wavelet-LLL_firstorder_Variance, wavelet-LLL_glcm_Autocorrelation wavelet-LLL_glrlm_ShortRunHighGrayLevelEmphasis
KNN	original_shape_Elongation, original_firstorder_Energy, log-sigma-0-5-mm-3D_glszm_LargeAreaEmphasis log-sigma-0-5-mm-3D_glszm_LargeAreaHighGrayLevelEmphasis log-sigma-1-mm-3D_firstorder_Energy, log-sigma-1-5-mm-3D_glszm_LargeAreaEmphasis log-sigma-1-5-mm-3D_glszm_LargeAreaHighGrayLevelEmphasis, log-sigma-2-mm-3D_firstorder_Energy log-sigma-3-mm-3D_glszm_LargeAreaHighGrayLevelEmphasis, wavelet-LLH_firstorder_Energy wavelet-LLH_glszm_LargeAreaEmphasis, wavelet-LLH_glszm_LargeAreaLowGrayLevelEmphasis wavelet-LLH_glszm_ZoneVariance,wavelet-LHL_firstorder_Energy wavelet-LHL_glszm_LargeAreaEmphasis, wavelet-HLL_firstorder_Energy wavelet-HLL_glszm_LargeAreaEmphasis, wavelet-HHL_glszm_LargeAreaHighGrayLevelEmphasis wavelet-HHL_glszm_ZoneVariance
SVM	original_shape_Elongation, original_shape_Flatness,original_shape_LeastAxisLength original_shape_MajorAxisLength, original_shape_Maximum2DDiameterColumn original_shape_Maximum2DDiameterRow, original_shape_Maximum2DDiameterSlice original_shape_Maximum3DDiameter, original_shape_MeshVolume original_shape_MinorAxisLength, original_shape_Sphericity,original_shape_SurfaceArea original_shape_SurfaceVolumeRatio,original_shape_VoxelVolume original_firstorder_10Percentile, original_firstorder_90Percentile log-sigma-1-5-mm-3D_glszm_LargeAreaHighGrayLevelEmphasis wavelet-LHH_glszm_ZoneVariance,wavelet-HHL_glszm_ZoneVariance wavelet-LLL_glszm_LargeAreaHighGrayLevelEmphasis
LDA	original_glrlm_GrayLevelNonUniformity, original_gldm_SmallDependenceLowGrayLevelEmphasis log-sigma-0-5-mm-3D_firstorder_90Percentile, log-sigma-0-5-mm-3D_glszm_LargeAreaEmphasis log-sigma-1-mm-3D_glszm_LargeAreaHighGrayLevelEmphasis log-sigma-1-mm-3D_glszm_SmallAreaLowGrayLevelEmphasis log-sigma-1-5-mm-3D_glszm_LargeAreaHighGrayLevelEmphasis log-sigma-1-5-mm-3D_glszm_ZoneVariance, log-sigma-2-mm-3D_glrlm_GrayLevelNonUniformity log-sigma-2-mm-3D_glszm_ZoneVariance, log-sigma-2-5-mm-3D_firstorder_Skewness log-sigma-3-mm-3D_glszm_LargeAreaEmphasis log-sigma-3-mm-3D_glszm_SmallAreaLowGrayLevelEmphasis wavelet-LLH_glcm_ClusterShade, wavelet-HHL_glcm_Imc1 wavelet-HHL_glszm_SmallAreaLowGrayLevelEmphasis, wavelet-HHL_glszm_ZonePercentage wavelet-HHH_glszm_ZonePercentage, wavelet-LLL_glrlm_GrayLevelNonUniformity wavelet-LLL_glszm_LargeAreaLowGrayLevelEmphasis
QDA	log-sigma-0-5-mm-3D_firstorder_90Percentile, log-sigma-1-mm-3D_glszm_SmallAreaLowGrayLevelEmphasis log-sigma-1-5-mm-3D_glszm_LargeAreaEmphasis, log-sigma-1-5-mm-3D_glszm_ZoneVariance log-sigma-2-5-mm-3D_glcm_ClusterProminence, log-sigma-2-5-mm-3D_glcm_ClusterShade log-sigma-2-5-mm-3D_glszm_LargeAreaEmphasis, log-sigma-3-mm-3D_glszm_LargeAreaEmphasis log-sigma-3-mm-3D_gldm_LowGrayLevelEmphasis, wavelet-LLH_firstorder_Variance wavelet-LLH_glrlm_GrayLevelNonUniformity, wavelet-LLH_glszm_SizeZoneNonUniformity wavelet-LHL_glrlm_GrayLevelNonUniformity, wavelet-LHH_gldm_DependenceNonUniformity wavelet-HLL_glrlm_LongRunLowGrayLevelEmphasis, wavelet-HLH_glrlm_GrayLevelNonUniformity wavelet-HLH_gldm_DependenceNonUniformity, wavelet-HHH_glrlm_RunLengthNonUniformity wavelet-HHH_glszm_LargeAreaHighGrayLevelEmphasis, wavelet-LLL_firstorder_90Percentile
Naive	original_shape_Elongation, original_shape_Flatness,original_shape_LeastAxisLength original_gldm_GrayLevelNonUniformity, original_gldm_LargeDependenceHighGrayLevelEmphasis log-sigma-1-mm-3D_firstorder_Variance, log-sigma-1-5-mm-3D_glcm_ClusterShade log-sigma-1-5-mm-3D_glszm_ZoneVariance, log-sigma-2-mm-3D_firstorder_Variance log-sigma-2-mm-3D_glszm_ZoneVariance, log-sigma-2-mm-3D_gldm_LargeDependenceHighGrayLevelEmphasis log-sigma-2-5-mm-3D_glcm_ClusterProminence, log-sigma-2-5-mm-3D_gldm_LargeDependenceHighGrayLevelEmphasis, log-sigma-3-mm-3D_glcm_ClusterProminence log-sigma-3-mm-3D_glszm_LargeAreaLowGrayLevelEmphasis log-sigma-3-mm-3D_gldm_LargeDependenceHighGrayLevelEmphasis wavelet-HLL_gldm_LargeDependenceHighGrayLevelEmphasis, wavelet-HHL_firstorder_Energy wavelet-HHH_glszm_LargeAreaHighGrayLevelEmphasis wavelet-LLL_firstorder_10Percentile

Table 6. Effect of training size on the prediction power of radiomics features.

**Nodule Image**	**Prediction Performance with Feature Fractioning (AUROC)**		
	25%	*50%*	*70%*
Target nodule	0.827±0.006	0.843±0.008	0.859±0.011
Context nodule	0.822±0.008	0.847±0.007	0.863±0.016
Combined	0.825±0.012	0.853±0.014	0.886±0.020

Table 7. Effect of training size on the prediction power of deep features.

**Nodule Image**	**Prediction Performance with Feature Fractioning (AUROC)**		
	25%	*50%*	*70%*
Target nodule	0.841±0.010	0.872±0.006	0.887±0.010
Context nodule	0.875±0.004	0.906±0.011	0.915±0.006
Combined	0.883±0.004	0.901±0.011	0.920±0.009

Table 8. Statistical comparison of AUROC curves between radiomic features and deep features for original as well as augmented feature sets. Significant differences were marked in bold.

**Feature Type**	**Radiomics vs. Deep Features (*p-value*)**	
	Imbalanced Sets	*Balanced Sets*
Target nodules	**0.0021**	**0.0032**
Context nodules	0.9477	0.2319
Combined	**0.0238**	**0.0115**

Table 9. Statistical comparison of AUROC curves between target, context, and combined hybrid poos for original as well as augmented feature. Significant differences were marked in bold.

**Feature Type**	**Hybrid Feature Sets (*p-value*)**	
	Imbalanced Sets	*Balanced Sets*
Target vs. Context	**0.0359**	**0.0153**
Target vs. Combined	0.4315	0.1276
Context vs. Combined	**0.0077**	**0.0005**
